# Conserved Cu-MicroRNAs in *Arabidopsis thaliana* Function in Copper Economy under Deficiency

**DOI:** 10.3390/plants8060141

**Published:** 2019-05-29

**Authors:** Muhammad Shahbaz, Marinus Pilon

**Affiliations:** Biology Department, Colorado State University, Fort Collins, CO 80523-1878, USA; ms@sppg.ca

**Keywords:** plastocyanin, photosynthesis, copper deficiency, Cu-microRNA, copper protein, target mimicry

## Abstract

Copper (Cu) is a micronutrient for plants. Three small RNAs, which are up-regulated by Cu deficiency and target transcripts for Cu proteins, are among the most conserved microRNAs in plants. It was hypothesized that these Cu-microRNAs help save Cu for the most essential Cu-proteins under deficiency. Testing this hypothesis has been a challenge due to the redundancy of the Cu microRNAs and the properties of the regulatory circuits that control Cu homeostasis. In order to investigate the role of Cu-microRNAs in Cu homeostasis during vegetative growth, we used a tandem target mimicry strategy to simultaneously inhibit the function of three conserved Cu-microRNAs in *Arabidopsis thaliana*. When compared to wild-type, transgenic lines that express the tandem target mimicry construct showed reduced Cu-microRNA accumulation and increased accumulation of transcripts that encode Cu proteins. As a result, these mimicry lines showed impaired photosynthesis and growth compared to wild type on low Cu, which could be ascribed to a defect in accumulation of plastocyanin, a Cu-containing photosynthetic electron carrier, which is itself not a Cu-microRNA target. These data provide experimental support for a Cu economy model where the Cu-microRNAs together function to allow maturation of essential Cu proteins under impending deficiency.

## 1. Introduction

Copper deficiency in plants leads to defects in photosynthesis, chlorosis, reduced respiration, and wilting of leaves. Copper is not a “mobile” element. This means that under impending deficiency, Cu is not efficiently transported from older tissues to newly developing leaves, which become chlorotic [1]. In green tissue, the majority of Cu is found in the chloroplast [2,3]. Plastocyanin (PC) is a blue Cu protein that mediates electron transfer from the cytochrome-*b_6_f* complex to photosystem I (PSI) in the thylakoid lumen of oxygenic photosynthetic organisms [4]. *Arabidopsis* has two genes for PC, *PC1 (PETE1)* and *PC2 (PETE2)*. The PC2 protein is more abundant and responsive to Cu [5,6]. PC2 accumulation is a consequence of protein stability due to cofactor presence and is not due to transcript abundance changes [6,7,8]. In higher plants, PC is the only protein that can accept electrons from the cytochrome-*b_6_f* complex and *Arabidopsis* mutants with insertions in both PC genes are seedling-lethal on soil [9,10]. Cu is also a cofactor of Cu/Zn superoxide dismutase (CSD) proteins that function in the metabolism of reactive oxygen radicals. In *Arabidopsis*, CSD2 is active in the plastids, and CSD1 is active in the cytosol [11]. Both CSD1 and CSD2 receive the Cu cofactor from a copper chaperone called copper chaperone for superoxide dismutase, CCS [12,13]. In mitochondria, copper is required for the function of cytochrome-*c* oxidase (COX), the proton-pumping terminal oxidase in the respiratory electron transport chain in the inner membrane [14]. Three Cu atoms are bound by the core COX subunits I, II, and III, which are encoded in the mitochondrial genome. The ethylene receptors in the endomembrane system are copper-binding proteins [15,16]. All remaining Cu proteins are most likely apoplastic. These include the plant-specific blue copper proteins called phytocyanins, which includes plantacyanin, apoplastic ascorbate oxidases, amine oxidases, and laccases [17,18,19,20,21,22,23,24]. The laccase family has 17 members in *Arabidopsis* [22], and for several of these, a role in lignification and secondary growth of the vasculature has now been established [25,26].

MicroRNAs that are regulated by Cu availability and that target mRNAs encoding for Cu proteins are called Cu-microRNAs [27]. The four Cu-microRNAs of *Arabidopsis*—*miR397*, *miR398*, *miR408*, and *miR857*—were first discovered in deep sequencing projects [28] and regulation of CSD1 and CSD2 by *miR398* was shown by Sunkar et al. [29]. It was later found that *miR398*, and thus CSD1, CSD2, and CCS, are regulated primarily by Cu levels [13,30]. In addition, *miR397*, *miR408*, *and miR857* regulate the abundance of other Cu proteins in *Arabidopsis*, specifically laccases and the secreted protein plantacyanin in response to Cu availability [31]. The Cu-microRNAs are in turn regulated via a Cu-responsive transcription factor called SPL7 (squamosa promotor binding protein-like7), which also regulates Cu assimilation [8,32,33,34]. Three of the four *Arabidopsis* Cu microRNAs (*miR397*, *miR398*, and *miR408*) are among the most highly conserved microRNAs in plants [35]. This conservation suggests an important function. Because of the strong link with Cu, it was hypothesized that the Cu-microRNAs function in the Cu economy. According to this idea, in plants, which have symplasmic connections that allow both the sharing of nutrients and communication via small RNAs, the regulation via microRNAs provides a mechanism to save Cu and to allow essential Cu protein maturation, such as PC and COX, in actively growing cells of a tissue during impending deficiency [27]. The hypothesis rests on three legs. Two well-supported tenets are the very tight bonding of Cu atoms to its ligands [36] and the presence of symplasmic connections between cells in plants. The third leg of the hypothesis is that effective signaling in and between cells via microRNAs works to signal Cu status and that it can indeed help to tune Cu protein expression. This third leg needs further experimental support. The possible role of Cu-microRNAs in the Cu economy is based mostly on correlative evidence with some more direct support in *Arabidopsis* and in poplar [30,31,33,34,37,38]. In fact, the idea that Cu delivery to PC is a priority is not supported by the observation that PC2 protein levels are strongly affected by Cu deficiency even if mRNA levels were not affected [6]. Do Cu microRNAs actually make a difference for Cu economy? To test this, we aimed to inactivate the conserved Cu-microRNAs (*miR397*, *miR398*, and *miR408*) and to analyze, especially under impending deficiency, the effects on growth and Cu allocation to abundant and essential Cu proteins, such as PC1 and PC2, which are not down-regulated via a microRNA. Because Cu deficiency affects flower and pollen development with several compounding effects, we limited our study to the vegetative shoot before flowering [31,39].

The Cu-microRNAs are under control of SPL7 [8,32]. Because in a *spl7*-loss-of-function mutant, Cu uptake is also defective, a good test of Cu-microRNA function requires that its regulation is uncoupled from SPL7. We have tested single microRNA loss-of-function mutants for *miR398a/c* and for *miR408* and found no discernable phenotypes and certainly no defect in PC maturation [3,13]. Similarly, *miR398* overexpression caused no severe phenotypes on soil or on agar media [13,40]. However, Zhang et al. have reported a small effect on PC accumulation due to altered miR408 expression in a hy5/spl7 background [34]. Due to properties of the SPL7-mediated system, changes in single Cu-microRNAs can be predicted to show attenuated effects on Cu homeostasis because the addition or elimination of Cu-binding targets will affect the Cu pool sensed by SPL7, which will reset the expression of Cu-microRNAs and Cu uptake systems [27]. Due to the “dampening” properties of the system, we predict that disruption of just one Cu-microRNA will have very small effects. Thus, perhaps all or the majority of the Cu-microRNAs must be perturbed simultaneously before strong effects on Cu homeostasis can be expected. We focused here on the conserved Cu-microRNAs. Each of *miR397* and *miR398* have multiple loci and thus combinations (crosses) of knock-out (KO) lines to inactivate all Cu-microRNAs are virtually impossible. We therefore used a target mimicry construct to simultaneously inhibit all the conserved Cu-microRNAs [41]. Target mimicry relies on the production of a “designed” RNA transcript, which is modified from INDUCED BY PHOSPHATE STARVATION1 (*IPS1*), a non-coding RNA in *Arabidopsis* that functions to sequester miR399, a microRNA that functions in the regulation of phosphate homeostasis [41]. The native *IPS1* transcript contains a microRNA target site for *miR399* that cannot be cleaved and that should sequester and inactivate the microRNA. The original mimicry target site in *miR399* can be modified to inhibit other microRNAs [41].

## 2. Results

In order to simultaneously inhibit all three Cu-microRNAs, we designed a tandem target mimicry strategy (see Supplementary Figure S1). In this approach we modified the native IPS1 sequence [41] by replacing the miR399 target sequence with three, in tandem, target sites for miR397, miR398, and miR408, respectively. The construct was placed under the control of a constitutive 35S-CaMV promoter. The three conserved Cu-microRNAs should be sequestered by the modified IPS1-Cu-microRNA tandem mimicry construct, which is not a target for microRNA-directed cleavage due to inserted mismatches at the predicted target cleavage sites.

**Figure 1:**
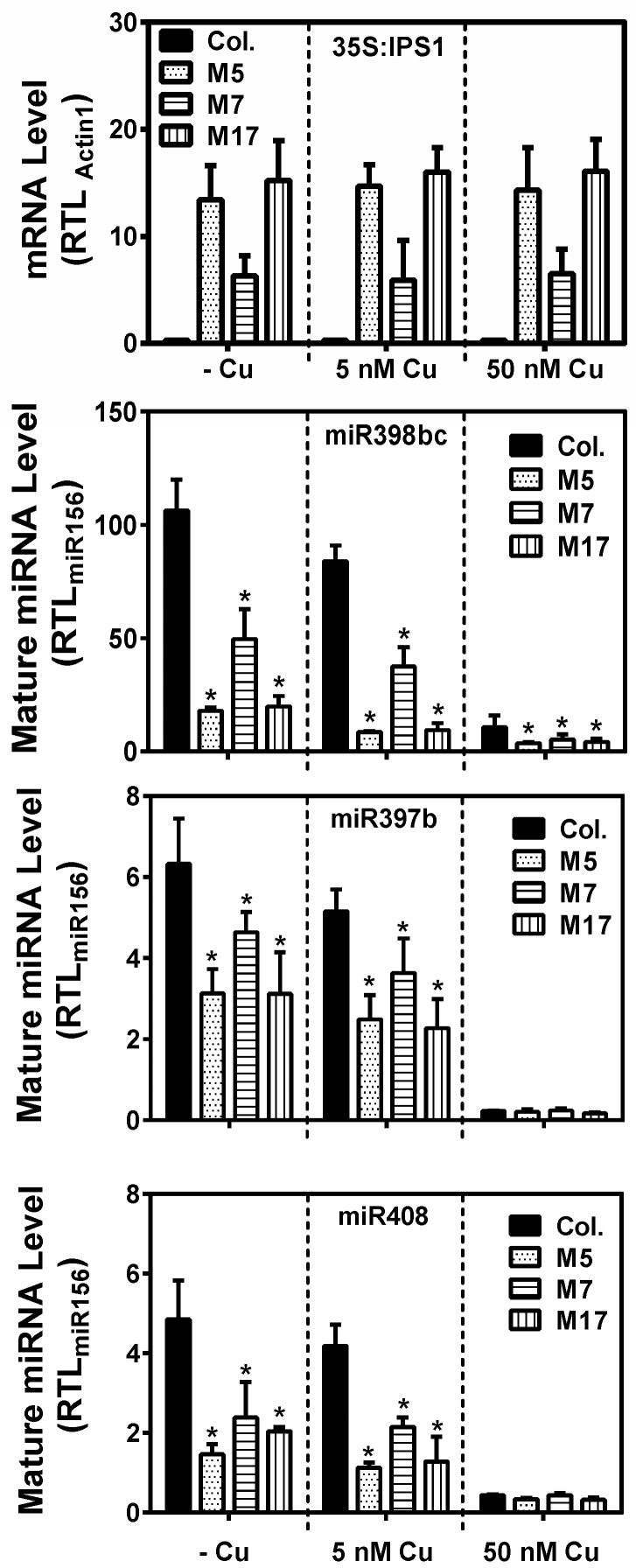
Expression of the IPS1 tandem target mimicry construct and three conserved Cu-microRNAs in wild type (Col) and three transgenic lines (M5, M7, and M17). 35S:IPS1 transgene relative transcript levels (RTL) were determined using qRT-PCR. Values are normalized relative to actin 1 expression. Mature microRNA levels were measured by qRT-PCR using primers designed for miR398bc, miR397b, and miR408. Relative microRNA levels were normalized to miR156 expression. All data and are given as averages ± SD (*n* = 3). * indicate significant differences from the control (*p* < 0.05, Student’s *t*-test).

Three transgenic *Arabidopsis* lines with high expression of the tandem mimicry construct were selected and compared to wild-type plants. To control Cu status, we germinated plants on Cu replete agar media for 10 days before transplanting the seedlings to hydroponics. We first verified that the expression in the shoot of the tandem mimicry construct was not affected by Cu-feeding status after 3.5 weeks in hydroponics (5 weeks after the start of germination) (Figure 1). We used three Cu regimes; no Cu addition for mild deficiency after 3–4 weeks in this growth condition; 5 nM CuSO_4_ for Cu-sufficient; and 50 nM CuSO_4_ for Cu-replete, a condition where all Cu proteins are expected to acquire this cofactor [31]. As expected, the target mimicry construct expression was not affected by Cu availability (Figure 1).

The tandem target mimicry construct was designed to affect the function of the three conserved Cu-microRNAs and prevent strong down-regulation of the transcripts for Cu proteins normally targeting the Cu-microRNAs, especially in low Cu conditions. We investigated, using quantitative reverse transcription polymerase chain reaction (qRT-PCR), the effect of the tandem target mimicry construct on the accumulation of the three conserved Cu-microRNAs at the three Cu concentrations (Figure 1). For both miR397 and miR398, three loci are present, whereas for miR408, a single locus exists. For miR397 and miR398, we utilized primers optimized to amplify the more abundant and Cu induced isoforms [8]. As expected, all three conserved Cu-microRNAs were strongly regulated by Cu availability in the wild-type, with highest expression seen when Cu was omitted from the growth medium (Figure 1). Conversely, in plants grown on 50 nM CuSO_4_, the expression of all three Cu-microRNAs was strongly repressed. Expression of the tandem target mimicry construct caused a notable reduction of all three Cu microRNAs. The effect on Cu-microRNA expression was more pronounced for miR398 and miR408 compared to miR397 (Figure 1). The effect of target tandem mimicry was most pronounced at 0 nM and 5 nM CuSO_4_, where in the wild-type, the Cu-microRNAs accumulated. Higher expression of the mimicry construct in lines M5 and M17 correlated with a stronger effect on Cu-microRNA accumulation.

**Figure 2:**
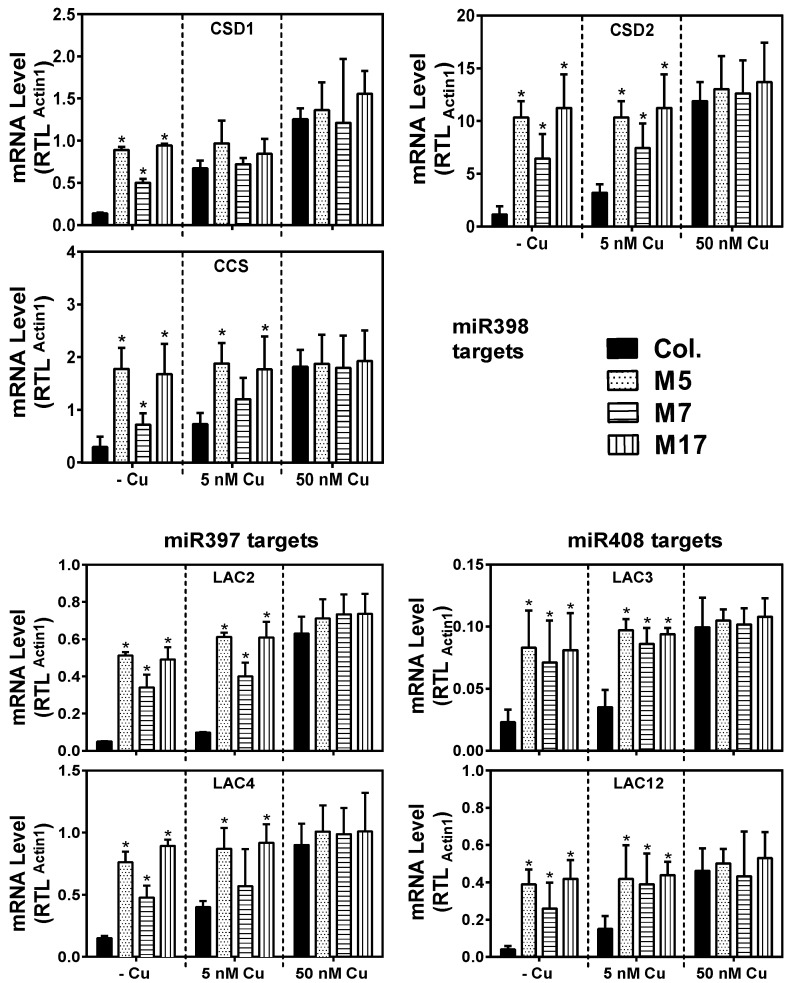
Expression analysis of selected Cu-microRNA target transcripts using qRT-PCR. mRNA-relative transcript levels (RTL) were normalized relative to actin 1 expression and given as averages ± SD (*n* = 3). * indicate significant differences from the control (*p* < 0.05, Student’s *t*-test).

We next analyzed the effect of tandem target mimicry on the transcript levels of selected confirmed Cu-microRNA targets (Figure 2). As expected, a strong effect of tandem target mimicry was seen for all target transcripts, this effect was most pronounced under Cu deficiency (- Cu). The deregulation by tandem target mimicry on Cu deficiency was strong for all the tested targets of the three conserved microRNAs (Figure 2). We conclude that accumulation of all the three conserved Cu-microRNAs was disrupted in the shoot by tandem target mimicry, and in turn this caused aberrant accumulation of transcripts encoding Cu proteins under Cu deficiency.

**Figure 3:**
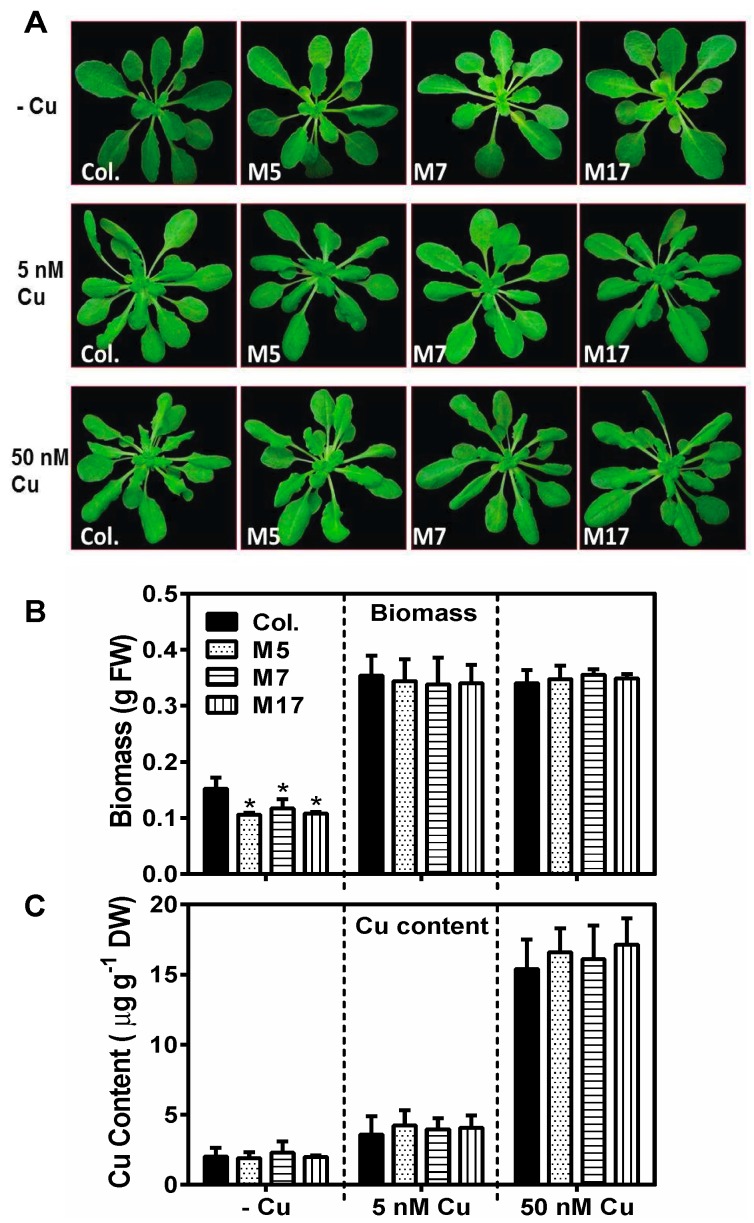
Effect of Cu-microRNA target mimicry on plant growth in hydroponics. (**A**) Representative images of 5-week-old WT (Col.); M5, M7, and M17 are All3-Cu miRNA mimicry lines. Plants were grown on 1/10th strength Hoagland solution containing 0, 5, or 50 nM CuSO_4_. (**B**) Fresh weight and (**C**) Cu content (µg g^−1^ DW) of 5-week grown plants. Values are given as averages ± SD (*n* = 6). * indicate significant differences (*p* < 0.05, Student’s *t*-test).

We next analyzed vegetative growth. Because the Cu-microRNAs are highly expressed in plants grown on low Cu and repressed in Cu-replete conditions, phenotypes due to target mimicry were mainly expected on low Cu conditions. Because we were especially interested in the effects on photosynthesis during vegetative growth, we compared plants at 5 weeks of age, which in our short-day growth condition is about a week before bolting, with the onset of symptoms of deficiency showing in the wild-type, without irreversible secondary effects. Because there is Cu in the seeds and in the germination medium, Cu depletion treatment in hydroponics takes several weeks to show visible deficiency symptoms. The plants of all genotypes showed overall comparable morphology on all three conditions after 3.5 weeks in hydroponics (Figure 3A). However, when the plant biomass was measured, a clear effect was seen for Cu omission compared to 5 nM and 50 nM CuSO_4_ (Figure 3B). Interestingly, in the deficient condition, a significant further reduction in biomass was seen for the three tandem target mimicry lines. We measured the elemental composition of the shoots. As expected, CuSO_4_ in the medium strongly affected Cu concentrations in the shoots. There was, however, no difference between the transgenics and wild-type, indicating that tandem target mimicry did not affect Cu levels (Figure 3C). Other elements did not show line-specific differences (See Table S1). In summary, tandem target mimicry for Cu-microRNAs resulted in a mild but significant effect on biomass accumulation, but only under Cu deficiency.

**Figure 4:**
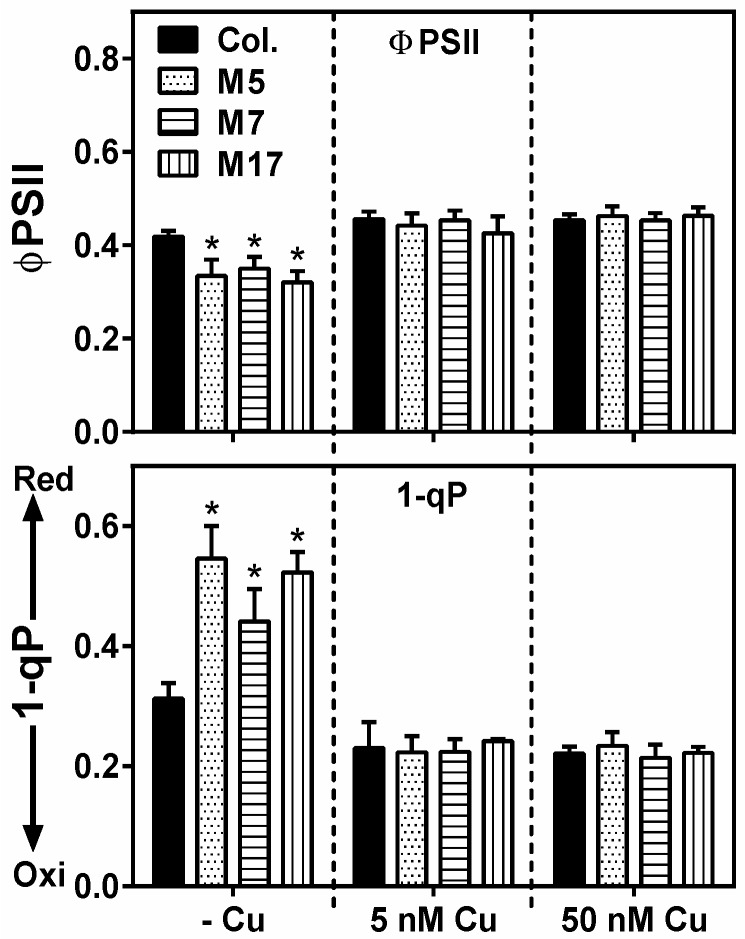
Photosynthetic electron transport parameters as measured by chlorophyll fluorescence. FluorCam measurement done on dark-adopted rosette leaves from WT (Col.) and transgenic lines (M5, M7, M17) growing in hydroponic culture at 0, 5, or 50 nM CuSO_4_, where values are given as averages ± SD (*n* = 6). Top panel: ΦPSII (estimate of electron transport). Bottom panel: 1-qP (indicative of the redox state of the plastoquinone pool). * indicate significant differences between lines within a growth condition (*p* < 0.05, Student’s *t*-test).

We hypothesized that tandem target mimicry, because of the misregulated and higher expression of Cu-microRNA targets, affected growth by limiting the pool of Cu available for plastocyanin, which is not a microRNA target. A lack of plastocyanin function should result in a decreased photosynthetic electron transport and a more reduced plastoquinone pool. Measurement of chlorophyll fluorescence parameters (Figure 4) indicated that Cu depletion in the wild-type caused a mild decrease in the flux through PSII (ΦPSII), which is an estimate of photosynthetic electron transport activity. However, there was a significantly larger decrease in ΦPSII for the three tandem target mimicry lines compared to the wild-type, but only under Cu deficiency. Similarly, the parameter 1-qP, which indicates the redox state of the plastoquinone pool, was affected by Cu depletion with a strong effect seen in tandem target mimicry. These results are strongly indicative of a defect in plastocyanin function in plants grown on low Cu, which is exacerbated by tandem target mimicry.

**Figure 5:**
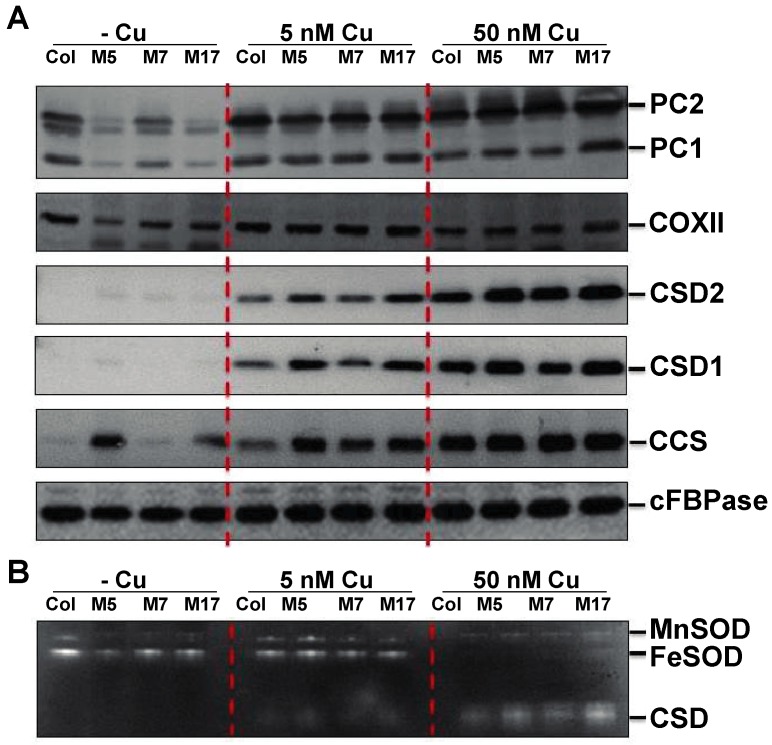
Comparison of Cu protein accumulation in wild-type (Col) and three transgenics (M5, M7, M17). (**A**) Western-blot analysis using the following antibodies: PC, plastocyanin two isoforms (PC2 and PC1 are detected); COXII, cytochrome-c oxidase subunit II; CSD2 and CSD1 (chloroplast and cytosolic isoforms of Cu/Z superoxide dismutase; and CCS, copper chaperone for Cu/ZnSOD. cFBPase was used as a loading control. Total soluble proteins (30 µg) were fractioned using SDS-PAGE (15% gel) and blotted onto nitrocellulose membranes. (**B**) For SOD isoform activity, total soluble proteins (30 µg) were fractioned on a non-denaturing 15% acryl amide gel and stained for SOD activity. The indicated detected isoforms were identified based on known relative mobility.

We next analyzed Cu protein accumulation using immunoblotting levels (Figure 5A). In *Arabidopsis*, two plastocyanin isoforms are expressed, PC1 and PC2 [10]. The more abundant PC2 isoform is known to be strongly affected at the protein level by Cu depletion, most likely due to a post-translational process as neither PC transcript is a target of a microRNA [6]. Indeed, Cu depletion in the wild-type resulted in a marked decrease in PC2 accumulation, while PC1 was relatively less affected (Figure 5A). Remarkably, there was a much stronger reduction in both PC isoforms in the three tandem target mimicry lines on low Cu. The severity of the effect on PC correlated strongly with the tandem target mimicry expression level (Figure 1 and Figure 5A). At 5 and 50 nM CuSO_4_, there was however no noticeable difference between the wild-type and transgenics. Cytochrome-c oxidase (COX) is a major Cu protein in the mitochondria where it functions as the terminal oxidase. We used antibodies specific to the Cu-binding COX core subunit II (COXII) as a proxy for COX accumulation (Figure 5A). COXII was affected by Cu depletion in the wild-type, albeit that the effect seemed not as strong as for PC in the chloroplast. There was a mild but noticeable larger effect of Cu depletion on COXII accumulation in the three target mimicry lines (Figure 5A).

We also verified accumulation of the miR398 targets: CSD1, CSD2, and CCS. As expected, accumulation of these proteins was strongly affected by Cu levels (Figure 5A). However, tandem target mimicry attenuated the effects of lowering Cu on the accumulation of these proteins. For CSD1 and CSD2 protein accumulation, the strongest effect of target mimicry was seen at 5 nM CuSO_4_, whereas for CCS protein accumulation, a strong effect was also seen at 0 nM CuSO_4_ (Figure 5A). The superoxide dismutase isozyme activity was analyzed using native gel assays (Figure 5B). As expected, the decrease in CSD1 and CSD2 protein levels resulted in a reduced CSD activity. FeSOD expression and activity, which is known to be highly Cu responsive and regulated via SPL7 [7,8,32], was not affected by tandem-target mimicry. In conclusion, lines that express the tandem target mimicry construct show a stronger reduction in plastocyanin and COXII levels on low Cu, which is accompanied by an elevated accumulation and activity of the miR398 targets.

## 3. Discussion

The conservation of the Cu microRNA sequences, their target transcripts encoding Cu proteins, and their regulation by Cu via SPL7 suggests an important function related to copper directly. Neither plastocyanin nor cytochrome-c oxidase core subunits are targets of a microRNA in plants, presumably because these proteins are indispensible. Could a role of the Cu-microRNAs be to maintain a pool of Cu accessible for plastocyanin and cytochrome-c oxidase? While such a role in the Cu economy has been proposed before, the direct evidence has been limited. We aimed to test a role of conserved Cu-microRNAs in the Cu economy in *Arabidopsis* under conditions that require photoautotrophic growth. In order to characterize a function of the conserved Cu microRNAs, we employed a tandem target mimicry strategy. Expression of target mimicry constructs caused a decrease in the microRNAs that were targeted (Figure 1). Such a decrease has been reported before for other microRNAs and indicates that target mimicry not only leads to target sequestration, but also causes increased microRNA instability. The Cu-microRNA deregulation was well correlated with the extent of mimicry construct expression. The tandem target mimicry approach clearly caused a deregulation of a large set of transcripts but (as expected) only on low Cu, a condition where Cu-microRNAs are highly expressed in the wild-type (Figure 1 and Figure 2). As a consequence of tandem target mimicry, the plants accumulated lower amounts of the photosynthetic electron carrier plastocyanin, one of the most abundant Cu proteins in plants. The exacerbated reduction in plastocyanin content in tandem target mimicry lines was strongly correlated with a defect in photosynthetic electron transfer and reduced biomass on low Cu (Figure 3, Figure 4 and Figure 5). These data provide strong and direct support for a role of the Cu-microRNAs in the Cu economy in *Arabidopsis*, requiring optimization of vegetative growth under low-Cu conditions. While the effect on growth was relatively small and required low Cu to be noted, we think it is likely a strong enough phenotype to explain the conservation of the Cu-microRNAs. In this context it should be noted that the non-conserved and fourth Cu-microRNA (miR857) was not targeted here [31]. Perhaps stronger phenotypes could be observed if this fourth target was also de-regulated.

Because of the presence of Cu in seeds and in the germination medium, which is needed to ensure comparable development, there is a significant lag time of about two and a half to three weeks before symptoms of Cu deficiency manifest themselves [31]. We wanted to avoid secondary effects, and therefore grew all plants for less than four weeks in hydroponics without Cu. We did this to avoid the compounding effects of bolting and flowering, which are strongly affected by Cu deficiency treatment, but only after strong and irreversible symptoms of Cu deficiency, such as leaf curling and browning, are evident in the vegetative shoot [31]. Therefore, in this study, we focused on vegetative growth and on shoots because this is where the Cu demand for plastocyanin is high. It seems likely, though, that the role of Cu-microRNAs, which are expressed in roots, stems, leaves, and flowers, is not limited to the vegetative shoot and indeed Cu feeding status is known to affect the timing of flowering, with deficient plants showing a delay [31]. Furthermore, Cu is also important for pollen development, presumably to allow for sufficient mitochondrial cytochrome-c oxidase activity [39]. We did not note any defect in fertility or seed set for our three target mimicry lines compared to wild-type. This was probably due to the sufficiency of Cu in the soil. It is possible that if the plants are to be subjected to Cu deficiency after the onset of flowering that a defect in pollen maturation would cause a decrease in fertility. However, such an experiment is difficult to control, and effects of both Cu and development would have to be considered simultaneously. Nevertheless, accumulation of mitochondrial COXII protein, one of three mitochondrial-encoded Cu-binding core subunits of cytochrome-c oxidase in the respiratory electron transport chain [14], was clearly negatively affected by tandem target mimicry in the shoots. This observation indicates that optimal cytochrome-c oxidase activity under Cu deficiency requires functional Cu-microRNAs. A role in regulating grain yield was also reported for miR408 in rice [42]. In this same study, overexpression of the rice miR408 target UCL8 (uclacyanin 8, a phytocyanin), showed a negative effect on plastocyanin and chloroplastic Cu accumulation in leaves [42].

In response to Cu availability, the Cu-microRNAs are regulated directly by SPL7, which is the plant homolog of the chlamydomonas (CRR1) copper response regulator [43]. SPL7 and CRR1 regulation requires *cis* elements called Cu-response elements (CuRe) with a GTAC core motif [43]. Thus, a strong mechanistic link exists for Cu and Cu-microRNA regulation. Another identified transacting factor that directly mediates miR408 Cu-microRNA expression via promoter area binding is HY5 (elongated hypocotyl 5). Co-regulation was shown for *miR408* by both SPL7 and HY5 [33,34]. This regulation makes sense since HY5 mediates gene expression in the light required for photosynthetic growth, which has a high demand for Cu. Plants that lack SPL7 or HY5 function had only a small defect in PC [34]. Overexpression of miR408 in the *spl7* and *hy5* mutants could, to some extent, alleviate this defect in PC maturation, albeit that plants were grown in vitro on agar media to see this effect [34]. Interestingly, miR408 overexpression could also rescue some developmental defects in *hy5/spl7* lines [34]. Therefore, a feedback loop was proposed as a mechanism to allow better PC maturation in miR408-overproducing lines, but it can also be argued that an improved Cu economy in such lines allows for better Cu cofactor availability to PC [34]. Consistent with this idea are the observations reported in several plant species where miR408 expression causes higher chloroplast Cu content and improved photosynthesis, growth, and seed yield in diverse plant species [42,44,45]. On the other hand, Carrió-Seguí et al. have reported that both overexpression and loss of miR408 caused a diminished plant performance, especially in low Fe conditions [46].

Besides Cu availability, other environmental conditions have been reported to affect Cu-microRNA expression [27]. This was convincingly shown in *Arabidopsis* for cold [47,48,49], Fe availability [46,50], or stresses that are predicted to induce reactive oxygen species accumulation such as high light, the herbicide methyl-viologen, and excessive toxic metal [29]. In our setup, we controlled growth conditions and attempted to avoid any additional stressors. It seems likely that Cu-microRNA expression is further tuned by environmental conditions apart from Cu and light. However, unlike for Cu and light, where *cis*-regions and interacting *trans*-acting factors (SPL7 and HY5) are identified, it is presently unclear at a mechanistic level how these conditions could be linked to gene expression of Cu-microRNAs. Therefore, the effects of other stresses on Cu microRNAs could be direct or indirect. For Fe, for instance, it is well-reported in the literature that lower Cu levels increases Fe uptake and vice versa. A mechanism for this interaction can be proposed based on the observation that both Fe and Cu uptake at the root require a reductase activity of the ferric reductase oxidase (FRO) family. A cross reactivity for the low Cu-induced FRO4/FRO5 with Fe, or for the low-Fe induced FRO2 with Cu, would lead to increased Cu uptake under Fe deficiency, thus resetting the SPL7 regulation of Cu microRNAs, making it appear as if Fe regulates Cu-microRNAs [8,51]. Therefore, to more directly link Cu-microRNAs to other abiotic stresses, it will be important to uncover how potential *cis*-regions in the promoters and transacting factors interact to mediate stress response.

## 4. Materials and Methods

### 4.1. Plant Material and Growth Conditions

Lines were propagated on PRO-MIX HP soil that was fertilized with Miracle-Gro Liquid All Purpose Plant Food (Scotts Company, Marysville, OH, USA). Wild type (WT; Col.) and All3-mimicry transgenic lines seeds were surface-sterilized and germinated on agar plates containing one-half strength Murashige and Skoog (MS) medium [52] supplemented with 1% sucrose. For hydroponics, 7–10 days old seedlings were placed on a one-tenth-strength Hoagland’s solution prepared with deionized water [53]. The nutrient solution was aerated and was replaced each week. For copper depletion (deficiency), Cu was omitted from the nutrient solution, while CuSO_4_ was added to 5 nM for low but sufficient Cu (sufficient for high PC activity, but with low CSD activity in the wild-type) and to 50 nM for the Cu-replete conditions (where all Cu proteins accumulate to maximum levels in the wild-type) [31]. To minimize Cu contamination, all containers and buckets used for Cu-deprivation had never been in contact with Cu. The plants were grown in a light intensity of 150 µmol m^-2^ s^-1^, with a 10-h-light/14-h-dark cycle and the temperature was maintained at 25 °C ± 2 °C in a climate-controlled room. After 4-weeks, plant material was harvested and stored frozen at −80 °C before analysis.

### 4.2. Transgenic Lines

Artificial target mimicry constructs were generated by modifying the sequence of the IPS1 gene [41]. The sequence of the construct, which was ordered as a synthesized piece of DNA (GenScript USA Inc. Piscataway, NJ, USA), is given in the Supplementary Materials. The All3-Cu miRNA target mimic construct was placed behind the CaMV 35S promoter in the pGWB41 vector, conferring resistance to kanamycin and hygromycin [54]. The construct was introduced into *A. thaliana* (accession Col-0) plants via a *Agrobacterium tumefaciens*-mediated transformation followed by selection on kanamycin containing agar media [55].

### 4.3. Elemental Analysis

For mineral content analysis, the plant tissue was placed in a drying oven at 55 °C for 48–72 h. One hundred milligrams of the dried material was digested in 1 mL of trace element grade nitric acid and heated at 60 °C for 2 h, followed by 130 °C for 6 h. The digests were subsequently diluted to 10 mL with double-distilled water before analysis using inductively coupled plasma – atomic emission spectrometry (ICP-AES) as described [56].

### 4.4. Chlorophyll Fluorescence Measurements

For chlorophyll fluorescence assays, whole rosettes of intact plants were used and dark-adapted for 30 minutes prior to analysis. Chlorophyll fluorescence imaging was done using a FluoroCam 701 MF (Photon Systems International, Brno, Czech Republic) at an actinic light intensity of 150 micro-Einsteins as described [13]. The parameters ΦPSII (flux through PSII, an estimate of photosynthetic electron the transport rate) and 1-qP (an estimate of the redox state of plastoquinone pool) were calculated as described [57].

### 4.5. Protein Accumulation

Soluble proteins were extracted as described [38]. Protein concentration was determined using the Pierce BCA protein assay kit (Thermo Scientific, Waltham, MA, USA) using bovine serum albumin as a standard. For western blotting, 20 µg of total protein was separated using 15% SDS-PAGE and then transferred onto a nitrocellulose membrane. Antibodies used for immunodetection of PC, CSD1, CSD2, and CCS have been described [13,37]. Antisera for COXII and cFBPase were obtained from Agrisera (Vannas, Sweden). All protein detection experiments were done at least in biological triplicate with comparable results, and representative gels are shown.

### 4.6. Quantitative Reverse Transcription-PCR

RNA from plants sampled in biological triplicate was isolated using the Trizol reagent following the manufacturer’s recommended protocol (Life Technologies, Carlsbad, CA, USA). After determination of total RNA concentrations, equal amounts per sample were reverse-transcribed using a First Strand cDNA Synthesis Kit (Life Technologies) and random hexamer primers. Quantitative RT-PCR was performed using the Light Cycler SYBR Green l master mix (Life Technologies) using gene-specific primer pairs as described previously for *Populus trichocarpa* using actin 1 as a housekeeping reference gene [37]. Samples without a template were used as negative controls. All primers are listed in Supplementary Table S2. qRT-PCR results and quality controls were analyzed using Light-Cycler 480 data-analysis software (version 1.5.1, Roche, Basel, Switzerland). The ΔΔCt method was used to calculated relative transcript expression levels.

### 4.7. Mature miRNA Stem-Loop qRT-PCR

For the quantification of mature microRNAs, a stem-loop pulsed RT was used [37]. The RNA was extracted using the Trizol method as described above; however, ethanol washes were avoided and nucleic acid precipitation steps were carried out after addition of 1/10th volume of sodium acetate (3 M; pH 5.2), followed by an equal volume of isopropanol [37]. The stem-loop pulsed RT and miRNA qRT-PCR were performed as described previously [37,58]. miRNA397b, 398b/c, and 408 abundance were analyzed using gene-specific primers, as described previously for *P. trichocarpa* [37]; see Supplementary Table S2). Relative mature miRNA abundance was standardized using miR156 expression [37]. Each sample was analyzed in biological triplicate.

### 4.8. Statistical Analysis

For statistical analyses the JMP software package (version 9.0.2; SAS Institute, Cary, NC, USA) was used. All results represent the averages and SD from at least three independent biological replicates. A Student’s *t*-test was used to calculate significant differences (*p* < 0.05).

## 5. Conclusions

Simultaneous inhibition of the function of three conserved Cu-microRNAs caused a mild but significant growth phenotype on low-Cu media, which can be ascribed to a lack of function of plastocyanin, which is essential for photosynthesis. These observations provide support for a function of Cu-microRNas is the Cu economy.

## Supplementary Materials

The following are available online at https://www.mdpi.com/2223-7747/8/6/141/s1. Figure S1: Conceptual model for tandem target mimicry, Table S1: Mineral composition of lines under three Cu regimes in mg/kg dw, Table S2: List of the primers used for qRT-PCR and mature miRNA stem-loop qRT-PCR.

## Abbreviations

CSD, Cu/Zn-superoxide dismutase; COX, cytochrome-c oxidase; PSII, flux through PSII; FeSOD, iron superoxide dismutase; 1-qP, redox state of plastoquinone pool; LAC, laccase; PC, Plastocyanin.
