# Supplementary material for: Conserved Cu-MicroRNAs in Arabidopsis thaliana Function in Copper Economy under Deficiency
Source: Plants (Basel). 2019 May 29;8(6):141. doi: 10.3390/plants8060141 (PMC6631055; doi:10.3390/plants8060141)
Supplement: Supplementary file 1 [file plants-08-00141-s001.pdf]

plants

Article

Conserved Cu-MicroRNAs in *Arabidopsis thaliana* Function in Copper Economy under Deficiency

Muhammad Shahbaz [1] and Marinus Pilon [2*]

Supplementary Materials.

Supplementary Materials: The following are available online at www.mdpi.com/xxx/s1, Figure S1: Conceptual model for tandem target mimicry, Table S1: Mineral composition of lines under 3 Cu regimes in mg/kg dw., Table S2: List of the primers used for qRT-PCR and mature miRNA stem-loop qRT-PCR.

Figure S1. Conceptual model for tandem target mimicry and construct sequence

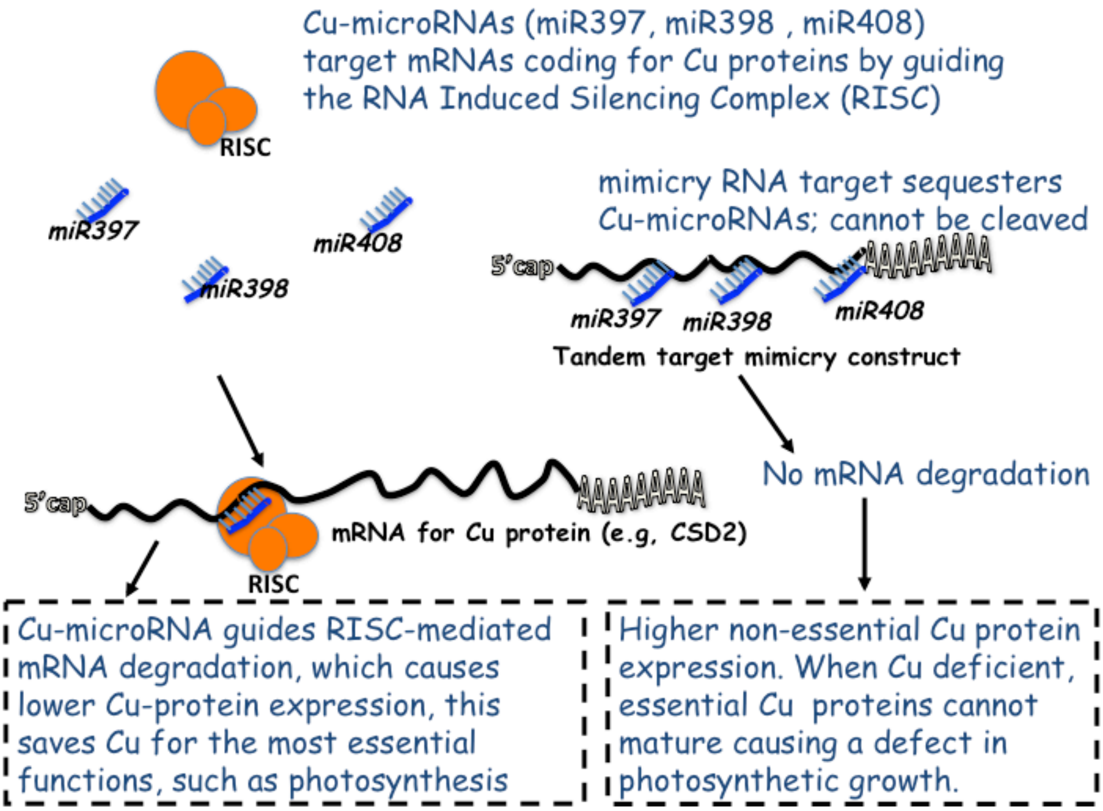

Tandem mimicry-construct sequence

gcggccgc ccatgg tctaga aaaacaccac aaaaacaaaa gaaaaatggc catcccctag ctaggtgaag
aagaatgaaa acctctaatt tatatagagg ttattcatct tttagcagta atgctactac actcaatgga
actctctaat taagtggttt tgtgttcatg taaggaaagc gttttaagat atggagcaat gaagactgca
gaaggctgat tcagactgcg agtttgtttt atctccctct agtaataggg gtgcctctat gagaacacat
gcttcggttc ccctcggaat cagcagatta tgtatcttta attttgtaat actctctctc ttctctatgc
tttgtttttc ttcattatgt ttgggttgta cccactcccg cgcgtgtcag ggacggctag gcagtgcata
aaaaatattc ggatttgaga actaaaacta gagtagtttt attgatattc ttgtttttca tttagtatct
aataagtttg gagaatagtc agaccggact ttgactctac gaatgtggat ctctttatag tgcattcctc
tt gagctc ggatcc gcggccgc

The modified target sequences for respectively miR397, miR398 and miR408 are highlighted. The construct is flanked at the 5′ end by *Not1*, *Nco1* and *Xba1* sites and at the 3′ end by *Sac1*, *BamH1* and *Not1* sites. The *Xba1* and *Sac1* sites were used for insertion into vector pGWB41.

Table S1 Mineral composition of lines under 3 Cu regimes in mg/kg dw

	**Col.**			**M5**			**M7**			**M17**		
**$CuSO_4$ Conc.**	**0 nM**	**5 nM**	**50 nM**	**0 nM**	**5 nM**	**50 nM**	**0 nM**	**5 nM**	**50 nM**	**0 nM**	**5 nM**	**50 nM**
**Ca**	37104 ± 1237	38136 ± 1999	38278 ± 1468	36203 ± 1429	40367 ± 544	39010 ± 1359	40112 ± 84	41312 ± 1155	49417 ± 1015	40156 ± 3924	39383 ± 317	41044 ± 61
**Fe**	42.1 ± 0.8	38.3 ± 3.6	37.6 ± 2.3	43.5 ± 1.9	40.1 ± 0.2	38.7 ± 0.4	43.7 ± 3.1	44.9 ± 3.1	45.9 ± 1.9	45.4 ± 1.2	38.9 ± 1.4	43.2 ± 2.5
**Mg**	3799 ± 4.5	3337 ± 223	3416 ± 164	3911 ± 188	3554 ± 30	3622 ± 68	4351 ± 93	3729 ± 57	3895 ± 4.9	4183 ± 10	3426 ± 47	3625 ± 22
**Mn**	33.2 ± 0.2	42.8 ± 1.8	37.6 ± 0.5	28.3 ± 2.2	42.8 ± 1.3	38.4 ± 2.6	33.1 ± 0.06	46.5 ± 0.6	42.4 ± 0.4	31.2 ± 1.4	44.6 ± 0.7	40.4 ± 1.8
**Mo**	4.3 ± 0.6	3.4 ± 0.2	2.8 ± 0.1	4.6 ± 0.1	3.2 ± 0.5	2.9 ± 0.1	4.2 ± 0.7	3.0 ± 0.2	2.7 ± 0.5	4.2 ± 0.4	3.5 ± 0.1	3.3 ± 0.1
**P**	9739 ± 174	9639 ± 805	9437 ± 699	8306 ± 269	10526 ± 48	9904 ± 213	9875 ± 584	11546 ± 144	10629 ± 791	9864 ± 764	10357 ± 32	9672 ± 11
**K**	27211 ± 655	30832 ± 2111	29761 ± 293	25209 ± 392	32027 ± 928	29900 ± 239	28480 ± 1278	34153 ± 1287	29510 ± 1542	28559 ± 230	32606 ± 48	30586 ± 15
**S**	8846 ± 458	8235 ± 873	7880 ± 931	7691 ± 279	8758 ± 452	8175 ± 327	8277 ± 327	8411 ± 1134	7675 ± 517	9696 ± 1678	8863 ± 428	8271 ± 104
**Zn**	42.1 ± 2.6	28.1 ± 2.5	34.8 ± 1.1	37.9 ± 1.8	29.1 ± 0.5	35.1 ± 2.7	46.3 ± 1.5	27.3 ± 1.2	37.3 ± 0.9	38.8 ± 2.7	30.1 ± 0.1	36.4 ± 0.2

Table S2, List of the primers used qRT-PCR and mature miRNA stem-loop qRT-PCR

**qRT-PCR primers**		
	Sequence (5' to 3')	Sense
CSD1	GCCTGGCTACTGGAAACGC	Forward
	GCTTAGTGTGGCTCAAAGCAT	Reverse
CSD2	CTCTAAGTCAGAGGCTAAGC	Forward
	CGTTTAAGTACATTTTGGTTT	Reverse
CCS	GTGATGGTACTGTCATATGGG	Forward
	CCAAAACTCTCTGTACTTTCA	Reverse
LAC2	GATGGTGAAACCTGGAAAGACG	Forward
	CGCTTCTACGACCGTCAATGT	Reverse
LAC3	TAGCCGCGGGTTATGGCAGC	Forward
	AGCCGGGTCTGTTCTCGGGT	Reverse
LAC4	CCATCTCAAGGTTACAAACTGTCAG	Forward
	CTTCTACCACCGTGAAAATATGGC	Reverse
LAC12	GCCCGATCCGACCCGGAAAG	Forward
	ATGAGGGCGGTTTGGCGGTC	Reverse
IPS1 tandem construct	GTTCCCCTCGGAATCAGCAG	Forward
	TTATGCACTGCCTAGCCGTC	Reverse
	**miRNA Stem-Loop qRT-PCR primers**	
	Sequence (5' to 3')	Sense
Reverse Transcription		
miR397-RT	GTCGTATCCAGTGCAGGGTCCGAGGTATTCGCACTGGATACGACCATCAA	Reverse
miR398bc-RT	GTCGTATCCAGTGCAGGGTCCGAGGTATTCGCACTGGATACGACCAGGGG	Reverse
miR408-RT	GTCGTATCCAGTGCAGGGTCCGAGGTATTCGCACTGGATACGACGCCAGG	Reverse
miR156a-g	GTCGTATCCAGTGCAGGGTCCGAGGTATTCGCACTGGATACGACGTGCTC	Reverse
qRT-PCR		
miR397b	GTGTGCCATTGAGTGCAGCG	Reverse
miR398b/c	TGGGTGTGTTCTCAGGTCG	Reverse
miR408	GTGTGATGCACTGCCTCTTC	Reverse
miR156a-g	ATGCGCTGACAGAAGAGAGT	Reverse
miRuniversal	CCAGTGCAGGGTCCGAGG	Forward
